# Severe acute respiratory syndrome coronavirus 2 infection and West Nile encephalitis in a patient with chronic kidney disease

**DOI:** 10.2478/jccm-2025-0040

**Published:** 2025-10-31

**Authors:** Vince Akos Andrejkovits, Alexandra Ioana Asztalos, Nina Ioana Bodnar, Erzsebet Iringo Zaharia-Kezdi, Anca Meda Vasiesiu

**Affiliations:** George Emil Palade University of Medicine Pharmacy Science and Technology of Targu Mures,Romania; Mures County Clinical Hospital, Targu Mures, Romania

**Keywords:** COVID-19, West Nile virus, encephalitis, end-stage renal disease, hemodialysis

## Abstract

**Objective:**

We describe a peculiar combination of West Nile virus (WNV) and SARS-CoV-2 infection, suggesting crucial clinical implications for diagnosis and management.

**Case report:**

We present a case of a 57-year-old woman with a past medical history of end-stage renal disease (ESRD), on chronic hemodialysis, and arterial hypertension. She was admitted to the hospital for a 5-day history of fever, headache, vomiting, psychomotor slowing, a diffuse tremor on the four limbs, and diarrhea. Evaluation revealed the presence of neutrophilic leukocytosis, hemoglobin level of 10.5g/dL, elevated C-reactive protein (60 mg/L), serum creatinine of 13.4 mg/dL with hyperkaliemia. Neurologic examination described the following findings: neck stiffness, confusion with motor aphasia, bradylalia, bradypsychia, global hyperreflexia, diffuse tremor, and unstable gait. Brain CT described a calcified temporo-lateral meningioma, CSF examination revealed colorless appearing, 560 leucocytes/3microL (97% lymphocytes), 848 mg/L proteins, glycorrhachia: 54 mg/dL (serum glucose: 101 mg/dL), and the multiplex Real-Time PCR test result was negative. On the second day of admission, the patient tested positive for COVID-19 and she was commenced on therapy with remdesivir, ceftriaxone, dexamethasone, and clexane. Adequate hemodialysis sessions were performed. On the eighth day of admission, the diagnosis of WNV infection was made based on the positive serological findings and the presence of IgM antibodies in the cerebrospinal fluid. After 15 days of hospitalization, the patient was discharged in good clinical condition, except for mild tremor in her limbs.

**Conclusions:**

Periodic epidemic bursts of WNV infection have been reported in Mures County, but present coinfection is rare; the severity and prognosis of the disease are unforeseeable.

## Introduction

In recent years, emerging infectious diseases have increasingly impacted human and animal health, posing significant global challenges for the health system and economy [[1,2]. About two-thirds of human diseases originate from wildlife, with many recent outbreaks caused by vector-borne viruses. Europe has identified at least ten mosquito-borne viruses (MBVs), with many belonging to the Flaviviridae family (West Nile, Dengue, and Usutu viruses). Other notable mosquito-borne viruses, such as Sindbis and Chikungunya, also have an important impact on public health [3,4].

West Nile Virus (WNV) is part of the Flavivirus genus and is the most prevalent mosquito-borne encephalitis virus. It has nine known lineages, with lineages 1 and 2 being the most common. Lineage 1 was isolated for the first time in South Africa in a female horse and her aborted fetus. However, lineage 5 was responsible for an outbreak of encephalitis in India in 1952, and is generally considered to be less virulent than other line-ages. Lineages 1 and 2 have been identified as the most implicated in human infections, including encephalitis [2,5,6]. WNV is primarily transmitted by *Culex mosquitoes* that feed on birds, which act as reservoir hosts [7]. Since its first detection in Albania in 1958, WNV has become endemic in Europe as well [8]. While most animals are not affected, humans and horses can become infected, though they typically do not transmit the virus back to mosquitoes. The majority of these infections are asymptomatic, but a small percentage of cases can result in neurological diseases. The virus also poses risks in the context of organ donation and blood transfusions, and mortality is higher in immunocompromised hosts [9,10,11].

The geographical areas most affected by WNV infections are those located in southern, southeastern, and central Europe. Warmer temperatures and recent climatic changes recorded in the past decades have allowed WNV to spread westward and to higher latitudes. The rapid spread of WNV lineage 2, initially identified in Hungary in 2004, has resulted in a substantial escalation of the infection in human and equine cases, particularly in southern and southeastern Europe [12].

In Romania, the most significant outbreak occurred in 1996, resulting in 393 hospital admissions and 17 fatalities. Since that time, annual cases have been documented, with notable outbreaks occurring in 2010, 2016, and 2018. WNV has been identified as endemic in southern and southeastern Romania, with cases also reported in the western regions, such as Sibiu, over the past decade [13,14].

Regarding the coinfection data between SARSCoV-2 and West Nile virus infection, we found only a few cases reported in the literature. A case report from Sacramento details the neurological onset of a 70-year-old female patient who was co-infected with COVID-19 and WNV. The patient’s condition was reported to have gradually improved [15]. Another co-infection was from the Luigi Sacco Hospital, Italy; this case concerned a 63-year-old woman with a history of systemic lupus erythematosus and lupus nephritis who was undergoing immunosuppressive therapy. The patient developed a fatal case of WNV meningoencephalomyelitis during her recovery from SARS-CoV-2. [16].

## Case Presentation

We describe a case of a 57-year-old female patient who presented at the hospital with fever (38.8 °C), headache, nausea, vomiting, semi-consistent stools, and an altered general condition. The present illness occurred within 72 hours. During the emergency department stay, there was noted deterioration in her health condition, resulting in the appearance of bradylalia, bradypsychia, and generalized tremor on the extremities, especially on the upper limbs.

There are no epidemiological data for recent travel or contact with individuals exhibiting similar symptoms. The patient’s previous medical history included the following: arterial hypertension, ESRD, on chronic hemodialysis, secondary anemia, and chronic cardiopathy. The patient is not an alcohol or tobacco/drug consumer, and there is a known history of ibuprofen allergy.

Laboratory tests performed at the emergency department revealed left shift with slightly increased C-reactive protein, elevated potassium, BUN (blood urea nitrogen), and creatinine (see Table I).

**Table I.** Laboratory results performed in the emergency department and during admission

**Test / Date**	**Before admission (emergency department)**	**12.08.2024**	**19.08.2024**	**23.08.2024**	**28.08.2024**
RBC (10^6^/µL)	3.62	3.35	3.36	3.39	2.98
Hematocrit (%)	34.6	32.3	32.1	31.6	28.8
Hemoglobin (g/dL)	11.8	10.80	10.5	10.6	9.4
Lymphocytes (%)	6	4.70	10.9	13.5	21.7
Lymphocytes (10^3^/µL)	0.92	0.66	0.87	0.76	0.82
Monocytes (10^3^/µL)	0.35	0.30	1.23	0.80	0.66
Monocytes (%)	2.30	2.20	15.4	14.1	17.5
Neutrophils (%)	91.6	93	72.5	70.1	
Leucocytes (10^3^/µL)	15.340	13.950	7.960	5.650	3.77
Platelet (10^3^/µL)	176.00	195.00	156.00	155.00	165.00
BUN (mg/dL)	70.62	154.08	119	76	92
Creatinine (mg/dL)	8.51	13.68	11.56	8.88	10.97
K (mmol/L)	6.27	6.64	4.21	4.75	6.2
Na (mmol/L)	140	142	138	144	139
CRP (mg/L)	24	-	8.82	16.29	3.24
Fibrinogen (mg/dL)	-	-	442.3	664.1	524.0
ESR (mm/1h)	-	-	52	78	-
INR	1.08	1.08	1.30	-	-
ALT (U/L)	22	14	20	-	13
AST (U/L)	20	17	14	-	19
Total bilirubin (mg/dL)	0.56	0.42	0.27	0.34	-
Serum glucose (mg/dL)	105	101	-	-	85

Chest and abdominal radiographs were not modified. SARS-CoV-2, Influenza virus A, and B rapid antigen tests yielded negative results. A native brain CT examination revealed a calcified meningioma measuring 7 mm on the left temporal side, with no additional pathological modifications.

A neurological evaluation revealed the presence of the following symptoms: bradylalia, bradypsychia, partial motoric aphasia, tremor of the limbs, and walking instability.

CSF analysis revealed a colorless appearance, 560 leucocytes/3microL (97% lymphocytes), 848 mg/L proteins, glycorrhachia: 54 mg/dL (serum glucose: 101 mg/dL), and the multiplex polymerase chain reaction (PCR) meningitis/encephalitis panel test was negative (see Table II for details). The smear and culture for *Mycobacterium tuberculosis*, from the CSF sample, returned a negative result. WNV-specific IgM testing was performed on serum and CSF.

**Table II.** Meningitis/encephalitis pathogen panel from cerebrospinal fluid sample

**Pathogen**	**Results**
***Escherichia coli K1***	Not detected
***Haemophilus influenzae***	Not detected
***Listeria monocytogenes***	Not detected
***Neisseria meningitidis (encapsulated)***	Not detected
***Streptococcus agalactiae***	Not detected
***Streptococcus pneumoniae***	Not detected
***Streptococcus pyogenes***	Not detected
***Mycoplasma pneumoniae***	Not detected
***Herpes Simplex Virus 1***	Not detected
***Herpes Simplex Virus 2***	Not detected
***Human Herpes Virus 6***	Not detected
***Enterovirus***	Not detected
***Human parechovirus***	Not detected
***Cryptococcus gattii/Cryptococcus neoformans***	Not detected
***Varicella-zoster virus***	Not detected

* QIAstat-Dx® Meningitis/Encephalitis (ME) Panel

She was admitted with the suspicion of acute meningoencephalitis to the 1st Infectious Disease Clinic of Târgu Mureș, Mureș County Hospital. The patient received antibacterial medication with Ceftriaxone and Vancomycin, in association with corticosteroids, depletive, and gastroprotectant. Hemodialysis sessions are conducted three times per week, accompanied by pre- and postdialytic laboratory tests (see Table III). The dosage of the treatment was modified in accordance with the presence of ESRD and chronic hemodialysis, in addition to the recommendations provided by the nephrologist.

**Table III.** The results of the blood tests taken before and after hemodialysis sessions

**Test / Date**	**2024-08-13**	**2024-08-13**	**2024-08-15**	**2024-08-15**	**2024-08-17**	**2024-08-19**	**2024-08-20**	**2024-08-21**	**2024-08-22**	**2024-08-23**	**2024-08-26**	**2024-08-28**
Creatinine (mg/dL)	14.63	6.54	12.41	5.33	6.56	11.56	13.24	10.52	13.28	8.88	10.97	10.97
BUN (mg/dL)	165	70	129	50	56	119	143	91	118	76	92	92
Potassium (mmol/L)	5.67	4.29	4.42	3.27	—	4.21	4.75	4.58	4.90	4.75	5.66	6.02
Sodium (mmol/L)	146	138	141	138	—	138	143	138	141	144	143	139

On the following days of hospitalization, the patient continued to receive antibiotic, anticoagulant, and steroidal anti-inflammatory treatment, despite the patient continues to present multiple low-grade fevers in a day, procalcitonin serum level was performed with a result of less than <0.5 micrograms/L, and blood cultures were collected (with negative results). The throat and nasal swabs, respectively, the sputum test was negative. Lingual secretion yielded *Candida non-albicans.* The treatment regimen comprised antibiotics (ciprofloxacin), antifungals, and antipyretics. The patient became afebrile after 48 h.

On the eighth day of the patient’s hospitalisation, the clinical symptoms of an acute onset upper respiratory tract infection were observed, accompanied by rhinorrhea and dry cough. A polymerase chain reaction (PCR) test was performed on a nasopharyngeal swab sample, the results of which indicated the presence of the SARS-CoV-2 virus. These findings supported the diagnosis of COVID-19.

The decision was taken to administer Remdesivir, with the addition of prophylactic anticoagulant (therapy using low molecular weight heparin). Thoracoabdominal-pelvic CT scan revealed the presence of left basal fibrotic bands, with no sign of pulmonary condensation or ground-glass opacity. The scan further demonstrates bilateral pleural effusion of approximately 6 mm, a hepatic cyst measuring 23/20 mm, and bilateral renal atrophy. EKG results were normal, echocardiography on cardiological evaluation ruled out the diagnosis of infective endocarditis, while describing a minimal pericardial effusion.

According to detailed anamnesis during admission, the patient reported multiple mosquito bites that occurred recently before the onset of symptoms. She was vaccinated against COVID-19 (two doses of the Pfizer-BioNTech vaccine).

On the ninth day of admission, the result of the WNV-specific IgM test in serum and CSF returned positive, thus confirming the diagnosis of *West Nile* meningoencephalitis. Antibiotic therapy was discontinued after 14 days; the patient exhibited a fever-free state, with reduced serum inflammatory markers.

The outcome during the hospitalisation was slowly favorable; the patient was discharged after 15 days, with improved neurological symptoms and general condition, with a mild residual tremor of the limbs.

## Discussions

The reported case of WNV meningoencephalitis along-side SARS-CoV-2 infection in a hemodialysis (HD) patient illustrates the multifaceted vulnerability of immunocompromised individuals.

As emphasized by Beck et al. and Och & Tylicki [15,17], patients with ESRD on chronic HD have significant immune dysfunction due to persistent inflammation, oxidative stress, and uremia. These factors compromise both innate and adaptive immune responses, thereby increasing the risk of community-and health-care-associated infections, including SARS-CoV-2, even in settings with high vaccination coverage. [18]. Studies have indicated that mortality is approximately four times higher among patients on dialysis than in the general population. It has been documented that the humoral immune response is diminished in patients undergoing HD after SARS-CoV-2 vaccination compared to healthy controls. However, the cellular immune response elicited by vaccination may be preserved in patients with ESRD. The benefits of vaccination outweigh the risk, and no dose reductions are recommended. Instead, the focus is on ensuring full vaccination coverage, possibly including an additional booster dose. Since 2023, the FDA and the EMA have not recommended reducing Remdesivir doses for patients with ESRD on hemodialysis with a standard regimen of a 200 mg intravenous (IV) loading dose on day 1, followed by 100 mg IV once daily for up to 5 days (can be extended to 10 in severe cases). Available data show that remdesivir is removed by hemodialysis, and no clinically relevant toxicity has been observed when standard doses are used [19]. In the case that has been presented, the role of vaccination was found to be of critical importance in mitigating the occurrence of severe outcomes associated with SARS-CoV-2.

WNV meningoencephalitis can be a potentially serious infection for individuals undergoing HD. This diagnosis may be suspected in cases of acute viral meningoencephalitis, particularly in elderly and/or immunocompromised patients from endemic regions, during summer and fall. In order to facilitate a timely and accurate diagnosis of meningoencephalitis, it is imperative to follow the guidelines for evaluating patients.

The outcomes of COVID-19 were associated with a high mortality rate among patients on chronic HD. The patient’s frequent exposure to healthcare services for dialysis raises the possibility of nosocomial COVID-19 acquisition, as mentioned in both studies discussed above [15,17]. Moreover, this case highlights the importance of implementing enhanced infection control protocols, developing comprehensive diagnostic approaches for neurologically symptomatic patients with ESRD, and implementing proactive strategies, such as booster vaccinations and early antiviral interventions, to reduce mortality in this particular group of patients.

In patients coinfected with both SARS-CoV-2 and arboviruses, the severity and prognosis of the disease are unpredictable. While both infections can cause mild illness, they have the potential to lead to severe, life-threatening disease in elderly and immunocompromised patients. A multidisciplinary approach comprising a neurologist and a nephrologist alongside an infectious diseases specialist is the most efficacious way of managing their coinfections in this vulnerable population. Such an approach promotes optimal recovery and prevents readmissions.

## Conclusion

Periodic epidemic bursts of WNV infection are reported in Mures County, but present coinfection is rare. The disease severity and prognosis of patients are difficult to predict, which poses significant challenges to diagnosis. A multidisciplinary approach has been shown to resolve issues and difficulties.
